# Developing and assessing a tool to measure motivation among physicians in Lahore, Pakistan

**DOI:** 10.1371/journal.pone.0209546

**Published:** 2018-12-31

**Authors:** Ahmad Azam Malik, Shelby Suzanne Yamamoto, Aminul Haque, Nadeem Shafique Butt, Mukhtiar Baig, Rainer Sauerborn

**Affiliations:** 1 Department of Family and Community Medicine, Faculty of Medicine in Rabigh, King Abdul Aziz University, Jeddah, KSA; 2 Institute of Public Health, University of Heidelberg, Heidelberg, Germany; 3 School of Public Health, University of Alberta, Edmonton, Canada; 4 Department of Population Sciences, University of Dhaka, Dhaka, Bangladesh; 5 Department of Biochemistry and Medical Education, Faculty of Medicine in Rabigh, King Abdul Aziz University, Jeddah, KSA; TNO, NETHERLANDS

## Abstract

Physicians’ motivation plays a vital role in health systems particularly in dense and urban cities, which deal with high volumes of patients in a variety of settings. The loss of physicians due to low motivation to developed countries is also a critical aspect affecting the quality of care in many regions. Fewer studies have explored health provider and particularly physicians’ motivation in developing countries, which is critical to health service delivery. In addition, limited relevant tools have been developed and tested in low and middle-income settings like Pakistan. The purpose of this study was to create and test a tool for measuring physician motivation. A tool was developed to explore physicians’ motivation in the Lahore district, Pakistan. Three sections of the questionnaire, which included intrinsic, socio-cultural and organizational factors, were tested with a stratified, random sample of 360 physicians from the public and private health facilities. Factor analysis produced six factors for ‘intrinsic motivation,’ seven for ‘organizational motivation’ and three for ‘socio-cultural motivation’ that explained 47.7%, 52.6% and 40.6% of the total variance, respectively. Bartlett’s test of sphericity and the KMO were significant. Cronbach's α and confirmatory factor analysis were found satisfactory for all three sections of questionnaires. In addition to identifying important intrinsic, socio-cultural and organizational factors study found the questionnaires reliable and valid and recommend further testing the applicability of the instrument in similar and diverse settings.

## Introduction

It has been estimated that the shortage of health care providers (HCP) is approximately 7.2 million, mostly in low and middle income settings of South Asia and Africa where the burden of disease is also high [1]. Undoubtedly, HCPs are one of the most critical facets of any health system [2, 3]. However, the needs of HCP are often not considered, especially in developing countries [4, 5]. Motivation is a critical aspect to consider for the achievement of any kind of health services targets or reforms [6]. Interestingly, motivation affects individual choices made according to goal-oriented behavior [7]. Unaddressed needs may manifest itself as low HCP motivation. Shortage of human resources and low staff motivation are considered to be major factors affecting health systems and health care particularly in developing countries [3]. Low motivation also is a strong push factor for the migration of HCP, usually from the regions where they are needed the most (e.g. from rural to urban areas or from developing to developed countries) [8] and may lead to loss of the already limited number of workers [9].

The terms of motivation and job satisfaction have been defined by scholars extensively over the last few decades. In general, literature is suggestive of multidimensional and inter-related facets of motivation and job satisfaction determinants. According to Luthans, motivation can be defined as the process that arouses, energizes, directs, and sustains behavior and performance [10]. Franco et al, defined motivation in work settings as "willingness to exert and maintain an effort towards organizational goals" [11]. Job satisfaction on the other hand is mentioned as “a pleasurable or positive emotional state resulting from the appraisal of one’s job or job experience” [12]. Largely, motivation is considered as the driving force to pursue and satisfy needs whereas job satisfaction is an emotional response to job conditions. Motivation and job satisfaction are distinct constructs but are known to be highly related, and often used interchangeably [13] and are highly interdependent to increase job performance in health care settings [14]. Gagne et al. explained self-determination theory and classified motivation into intrinsic and extrinsic motivation. Intrinsic is related to interests and spontaneous satisfaction as compared to extrinsic that relates different activities with separable outcomes or consequences [15, 16]. While few other scholars in relevant literature explores motivation determinants at intrinsic, organizational and socio-cultural factors [5, 11]. In addition, two well-known content theories, Maslow's need-hierarchy theory and Herzberg's two factor theory, which influenced the choice of items in the measuring instrument used in this study are briefly described in methodology section.

Unfortunately, though research on HCP has been conducted extensively in developed countries, little has been accomplished in developing countries [11, 17, 18]. At present, there have been a few studies conducted in Africa [18–20], but the literature is notably scarce in Asia [21] and particularly from Pakistan [22]. Rather than using existing developed country instruments as templates that can be modified to fit developing countries, it has been suggested that new tools be created that incorporate the intrinsic, organizational and socio-cultural realities of HCP working in these areas [18]. Different circumstances necessitate the development of different tools, sensitive to social, cultural, organizational and population differences. Studies have frequently used psychological tools to explore HCP motivation in developed countries, but relatively less is explored in developing countries [21, 23]. Limited studies have explored motivation and/or job satisfaction among health workers in Pakistan. Interestingly, a study on public sector workers found low level of motivation [22] while another study found that low motivation level produces high stress at work [24]. Physician motivation, specifically, is an understudied but crucial aspect of health care provision as it strongly affects issues such as patient outcomes and resource utilization, among others. Therefore, the purpose of this study was to create and test a tool for measuring physician motivation in Pakistan and assist in filling this gap.

## Materials and methods

The overall study consisted of three components. Part one was comprised of semi-structured, open-ended questions about physicians’ work motivators and demotivators published elsewhere [25]. The second part consisted of a tool of three sections of the questionnaire with closed-ended questions that used Likert scales for the quantification of factors related to motivation. A third part involved 16 in-depth, one-on-one interviews with physicians planned to be published elsewhere. The results presented here are from second part of the study and mainly focus on the development and testing of the tool used to assess motivation quantitatively.

### Study location

The study was conducted in the densely populated district (4681 persons/km^2^) of Lahore, (capital of Punjab province), Pakistan [26]. The existing health system infrastructure in Pakistan consists of three tiers. These include Basic Health Units (BHUs), Rural Health Dispensaries (RHDs) and Rural Health Centers (RHCs) as the major primary facilities. Secondary facilities include both primary and secondary referral facilities that provide acute, ambulatory and inpatient care through Tehsil Headquarter Hospitals (THQs) and District Headquarter Hospitals (DHQs). Tertiary facilities are large, major teaching hospitals and institutes (both public and private) with specialized wards and treatment programs. The Lahore district has 37 BHUs, six RHCs and 23 RHDs at the primary level, two Tehsil and two District Headquarter Hospitals at the secondary level and eight private and four public medical institutes with their affiliated teaching hospitals at the tertiary level. Primary and secondary facilities fall mainly under the responsibility of district governments. Tertiary facilities usually are administered by the provincial governments. Private sector facilities are poorly regulated, and no actual controlling body exists, resulting in varying standards of care. In general, Pakistan has struggled to counter the scarcity of resources, along with under-productivity, mal-distribution, migration and social threats to health workers and despite given much attention to increasing the number of doctors and medical schools it still falls well short of international recommendations [27]. With approximately 6,800 medical students graduating annually, the number of younger physicians is expected to rise in the coming years. Nevertheless, this age group has a greater chance of migrating to a developed country or from rural to urban areas. In Punjab physicians to population ratio is even worst around < 0.2/1000 population that is further burdened by around 100–1500 physicians leaving the country annually [27].

### Tool development

**Fig 1:**
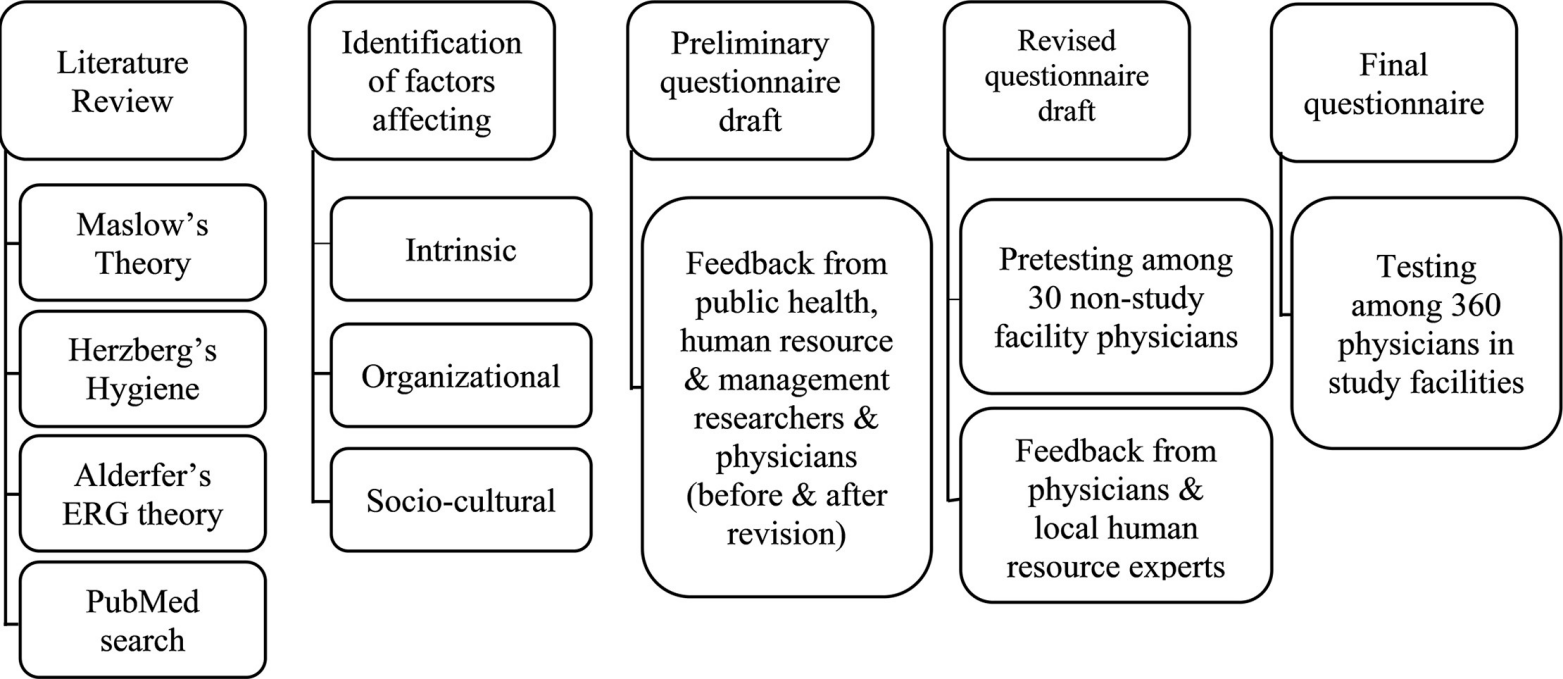
Summary of tool development and testing process.

Tool development and testing steps are outlined in Fig 1. Items were prepared in English. Conscientious consideration was given to item phrasing to create an understandable and concise instrument that would achieve high response rates and reduce the possibility of biased results. All sections of questionnaire were developed using information from the literature and existing tools.

Tool was aimed to be designed for health providers’ motivation particularly in developing countries. A review of the literature related to our study objectives (physicians’ satisfaction and motivation) was also conducted. We used the search terms “health provider”, “motivation”, “job satisfaction” and “tools” in PubMed to search for articles with these keywords included in the title or the abstract. We also combined and used Medical Subject Heading Terms (MeSH) terms and snowballing to broaden our literature search. Specific inclusion criteria (peer-reviewed publications in English) were set during the literature search in order to identify both emerging and established motivational factors, which could be included in the questionnaire.

In order to make it a better fit with existing literature, extensive literature review was conducted that included studies from developed and developing countries as well as theories and conceptual frameworks. In addition related reviews were taken into consideration [5, 28–30]. Studies reviewed on multidimensional job satisfaction and/or motivation provided the support in tool development. Many studies were considered relevant to identify questions, constructs and categories [4, 6, 11, 31–43]. Additionally, relevant theories [16, 44–46] and conceptual frameworks [11, 31, 32, 47, 48] were found relevant and further reviewed for guidance so as to better align tool in theses perspectives. Maslow’s theory explained individual needs in terms of a hierarchy: physiological needs as the most basic followed by security or safety needs, social needs, esteem needs and the need for self-actualization, in ascending order. When a need or set of needs is substantially fulfilled, that need ceases to be a motivator, and the next takes its place [46]. Although research and literature show scarce practical verification and validation of Maslow’s theory, its perceptive reasoning and simplicity have led to its broad appreciation and application [49]. In an attempt to transform Maslow’s theory, Herzberg developed the Hygiene theory suggesting that intrinsic factors are associated with satisfaction, while extrinsic factors with dissatisfaction. Applying this to motivation, the existence of certain factors (security, status, relationships with co-workers, personal life, salary, work conditions, company policy and administration) in the workplace may not necessarily lead to motivation, though their absence may produce demotivation. Similarly, while the deficiency of some factors (growth, job advancement, responsibility, challenges, recognition, and achievements) may cause no dissatisfaction, their presence may motivate and satisfy workers [45, 50]. A study among nurses in the US found evidence to support Herzberg’s theory [51]. However, little is known about the relative importance of these various determinants in developing country contexts. Alderfer also modified Maslow’s needs theory into the ERG or Existence, Relatedness and Growth theory [44]. Existence includes physiological and safety needs, Relatedness consists of social and external esteem needs and Growth includes internal esteem and self-actualization. Alderfer also suggested that more than one need may be driving an individual at one time.

Motivational determinants are usually classified as intrinsic or extrinsic factors[15, 16]. In order to examine certain motivators in more detail, we further divided extrinsic factors into organizational and socio-cultural factors [11, 14, 21, 25, 52] Consequently, several factors including both financial and non-financial were identified. Factors were later categorized into intrinsic, organizational and socio-cultural categories [5, 11]. The initial questionnaire draft included background information of study population such as; age, gender, marital status and working hours per week, 35 intrinsic (e.g. basic needs), 63 organizational (e.g. working conditions) and 18 socio-cultural (e.g. social rewards) items. A five-point Likert scale was provided for the responses (strongly agree to strongly disagree).

### Pretesting of the questionnaires

Probably due to complex and dynamic nature of motivation and job satisfaction many factors were overlapping in various settings. Therefore, for content validity local HR and psychometric experts and physicians were consulted throughout the tool development process to cover relevant theoretical framework, keep comprehensive approach, reduce overlap and bias and have appropriate questions, constructs and later categories that better suits the local study settings. Questionnaire was also reviewed for its clarity, entirety for appearance, item sequence and completion time.

After the identification and removal of unclear, overlapping, and irrelevant questions, the final set of structured, self-administered questionnaire showed Cronbach’s α value of 0.88 from sample of 30 in pretesting. Three sections finalized for the study consisted of 22 items for ‘intrinsic motivation’, 36 items for ‘organizational motivation,’ and 15 items for ‘socio-cultural motivation’ showed Cronbach’s α values of 0.75, 0.87 and 0.65 respectively.

### Testing of the final questionnaire

The instrument was then administered to a stratified random sample of 360 physicians from 1406 total physicians representing all the public primary and secondary health facilities and two tertiary level facilities (one private and one public) having the largest number of physicians, were included [25].An equal number of male and female physicians were chosen from each stratum. All medical practitioners registered with the Pakistan Medical and Dental Council working in the study health facilities during the time of recruitment were eligible for the study.

### Ethics, consent, and permissions

Ethical approval for the study was obtained from the Ministry of Health, Punjab, Pakistan and the Ethical Committee of the Medical Faculty, University of Heidelberg, Germany. Consent was obtained from all of the participants involved, for participation and to further disseminate study findings as for any scientific format.

### Statistical analysis

Quantitative data were analyzed by exploratory factor analysis with varimax rotation using IBM-SPSS and AMOS version 21 to determine the construct validity of each section of the questionnaire [53]. The purpose was to group related items according to common themes or factors, eliminate redundant items and identify those items that were related to more than one factor. Scree tests and eigenvalues were also considered in the selection of items [53, 54]. Factorability of the correlation matrix (R) was evaluated using Bartlett’s test of sphericity and Kaiser-Meyer-Olkin’s (KMO) measure of sampling adequacy. For items that cross-loaded on two or more factors, the background and meaning of the item and/or loading weight were used in factor assignment. Factor loadings greater than 0.40 were judged to be meaningful [55, 56]. The internal reliability and consistency of all sections of the questionnaire were measured using Cronbach’s α. After conducting EFA, construct validity was further assessed by confirmatory factor analysis (CFA) to confirm factor model. Modification indices were consulted to determine if there was an opportunity to improve the model.

## Results

### Descriptive statistics

Background information about the study population is presented in greater detail elsewhere [25]. Briefly, physicians ranged in age from 23 to 49 years, the majority of whom (71.9%) were ≤ 30 years. Most of the physicians that participated were single (57.2%). The mean working hours of physicians per week and years at their current health facility were 56.9 hours and 2.5 years, respectively.

### Assessment of questionnaire

#### Exploratory Factor Analysis (EFA)

We conducted an EFA using Maximum Likelihood with Varimax rotation to see if the observed variables loaded together as expected, were adequately correlated, and met criteria for reliability and validity [53]. Cronbach’s α value of 0.89 was found for the whole questionnaire. When explored for each section separately, values of 0.65, 0.88 and 0.60 were observed for intrinsic, organizational and socio-cultural sections respectively. Questionnaire as well as its all three sections (Intrinsic, organizational and socio-cultural) when explored for adequacy, were found significant p < 0.001 and demonstrated sufficient convergent & discriminant validity with recommended minimum threshold [55].

The final factor analysis of questionnaire items produced six factors for ‘intrinsic motivation’, seven for ‘organizational motivation,’ and three for ‘socio-cultural motivation.’ For intrinsic motivation, seven factors were extracted initially with eigenvalues ≥1, which explained 50.7% of the total variance. To obtain more parsimonious results, the factor “Work independence”, which had only one item, was excluded leaving 21 items (Table 1). The final six factors (i.e. those with scientific value, significance, meaning and consistency) explained 47.7% of the total variance.

**Table 1:** Intrinsic factors.

Rotated Component Matrix						
**1**	**Basic needs**	**1**	**2**	**3**	**4**	**5**	**6**
1.1	Availability of safe drinking water	**.863**					
1.2	Availability of adequate food	**.847**					
1.3	Availability of proper rest rooms	**.694**					
**2**	**Level of work interest**						
2.1	Interest in current job		**.747**				
2.2	Like my work		**.592**				
2.3	Work is meaningful to me		**.564**				
2.4	Challenging work is given to me		**.534**				
**3**	**Personal values**						
3.1	Work as a source of social respect			**.739**			
3.2	Self-respect from work			**.652**			
3.3	Able to work ethically, in general			**.530**			
3.4	Sense of accomplishment during work			**.481**			
**4**	**Work pride and internal recognition**						
4.1	Status in the hospital				**.754**		
4.2	Importance given to me by hospital management				**.653**		
4.3	Pride that I receive being a part of this organization				**.549**		
**5**	**Self-actualization and esteem**						
5.1	Variety in my activities at work					**.684**	
5.2	Opportunities for creativity at work					**.669**	
5.3	Recognition of good work					**.525**	
**6**	**Intrinsic satisfaction**						
6.1	Feel dependable and reliable						**.741**
6.2	Like to work hard						**.522**
6.3	Work satisfaction, in general						**.463**
6.4	Work efficiency						**.411**

For ‘organizational motivation,’ factor extraction initially gave eight factors with eigenvalues ≥1, which explained 53.0% of the total variance. An item that loaded less than 0.40 and one unnamed factor was deleted, which included the two unrelated items (career growth and workload) leaving 33 items to obtain final seven-factor solution (Table 2) which explained 52.6% of the variance.

**Table 2:** Organizational factors.

	Rotated Component Matrix							
**1**	**Working conditions and facilities**	**1**	**2**	**3**	**4**	**5**	**6**	**7**
1.1	Availability of proper dressing rooms	**.786**						
1.2	Availability of proper toilets and hand washing facilities	**.782**						
1.3	Availability of appropriate environment for treating patients	**.775**						
1.4	Availability of proper lighting during work	**.715**						
1.5	Availability of clean and maintained workplace	**.682**						
1.6	Availability of adequate, designated work area	**.681**						
1.7	Availability of proper ventilation in workplace	**.673**						
1.8	Satisfaction with the general work environment	**.579**						
**2**	**Job roles and responsibilities**							
2.1	Satisfaction with clarity of my job description		**.675**					
2.2	Satisfaction with referral procedures		**.648**					
2.3	Satisfaction with work according to my job description		**.635**					
2.4	Satisfaction with clarity of my roles and responsibilities		**.571**					
2.5	Satisfaction with clarity of roles and responsibilities of other HCPs		**.528**					
**3**	**Work related health and safety**							
3.1	Satisfaction with safety during work from diseases			**.681**				
3.2	Satisfaction with personal safety and security measures at work			**.611**				
3.3	Satisfaction with occupational health and safety measures in general at work			**.577**				
**4**	**Pay**							
4.1	Good competitive salary for this profession				**.715**			
4.2	Income is in accordance to my education, skills, performance and knowledge				**.688**			
4.3	Salary is enough to fulfill my and my family’s basic needs				**.687**			
4.4	Satisfaction with salary increments				**.509**			
**5**	**Incentives other than salary**							
5.1	Satisfaction with pension plan					**.665**		
5.2	Satisfaction with allowances					**.648**		
5.3	Satisfaction with opportunities for higher qualification					**.574**		
5.4	Satisfaction with opportunities for promotion					**.521**		
5.5 5.6	Satisfaction with job security Satisfaction with insurance plan					**.476** **.403**		
**6**	**Resource availability**							
6.1	Satisfaction with supplies available at work						**.619**	
6.2	Satisfaction with equipment available at work						**.617**	
6.3	Satisfaction with drugs available at work						**.601**	
6.4	Satisfaction with appropriate number of staff available at work						**.574**	
**7**	**Supervision**							
7.1	Satisfaction with feedback received from my supervisor							**.744**
7.2	Satisfaction with quality of supervision received							**.712**
7.3	Satisfaction with supervisor feedback							**.704**

The initial factor extraction for ‘socio-cultural motivation’ gave five factors with eigenvalues ≥1, which explained 48.7% of the total variance. Two factors with less than two items, “Teamwork” with two items and an item “Work affects other priorities like responsibilities at home, visiting friends, pursuing a hobby, etc.” were deleted leaving 12 items. The final analysis provided three factors (Table 3), which explained 40.6% of the variance. All factors were labeled after exploration of the items.

**Table 3:** Socio-cultural factors.

	Rotated Component Matrix			
**1**	**Work-related interpersonal relationships**	**1**	**2**	**3**
1.1	Co-workers help each other at work	**.668**		
1.2	Co-workers respect each other	**.646**		
1.3	General interpersonal relations at work	**.594**		
1.4	Relationship between doctors and nurses	**.481**		
1.5	Co-workers willingly share expertise and skills with other colleagues	**.432**		
**2**	**Social recognition**			
2.1	Respect I receive from the patients		**.710**	
2.2	Feedback I receive from patients		**.653**	
2.3	Respect I receive from the community		**.612**	
2.4	Feedback I receive from the community		**.402**	
**3**	**Personal life issues**			
3.1	Personal support from other HCPs when required			**.669**
3.2	Satisfaction with my personal life issues			**.641**
3.3	My hospital supports and respects my personal life issues			**.548**

#### Determinants of motivation

For ‘intrinsic motivation’ the six factors were basic work needs [three items], personal values and ethics [four items], work interest and importance [four items], work pride and internal recognition [three items], self-actualization and esteem [three items] and intrinsic satisfaction and self-sufficiency [four items]. For ‘organizational motivation’, the seven factors (33 items) were labeled as working conditions and facilities (eight items), job roles and responsibilities (five items), work-related health and safety (three items), pay (four items), incentives other than salary (six items), resource availability (four items), and supervision (three items). For ‘socio-cultural motivation’ three factors (12 items) were labeled as work-related interpersonal relationships (five items), social recognition (three items) and personal life issues (four items).

### Confirmatory Factor Analysis (CFA)

#### Model fit

**Fig 2:**
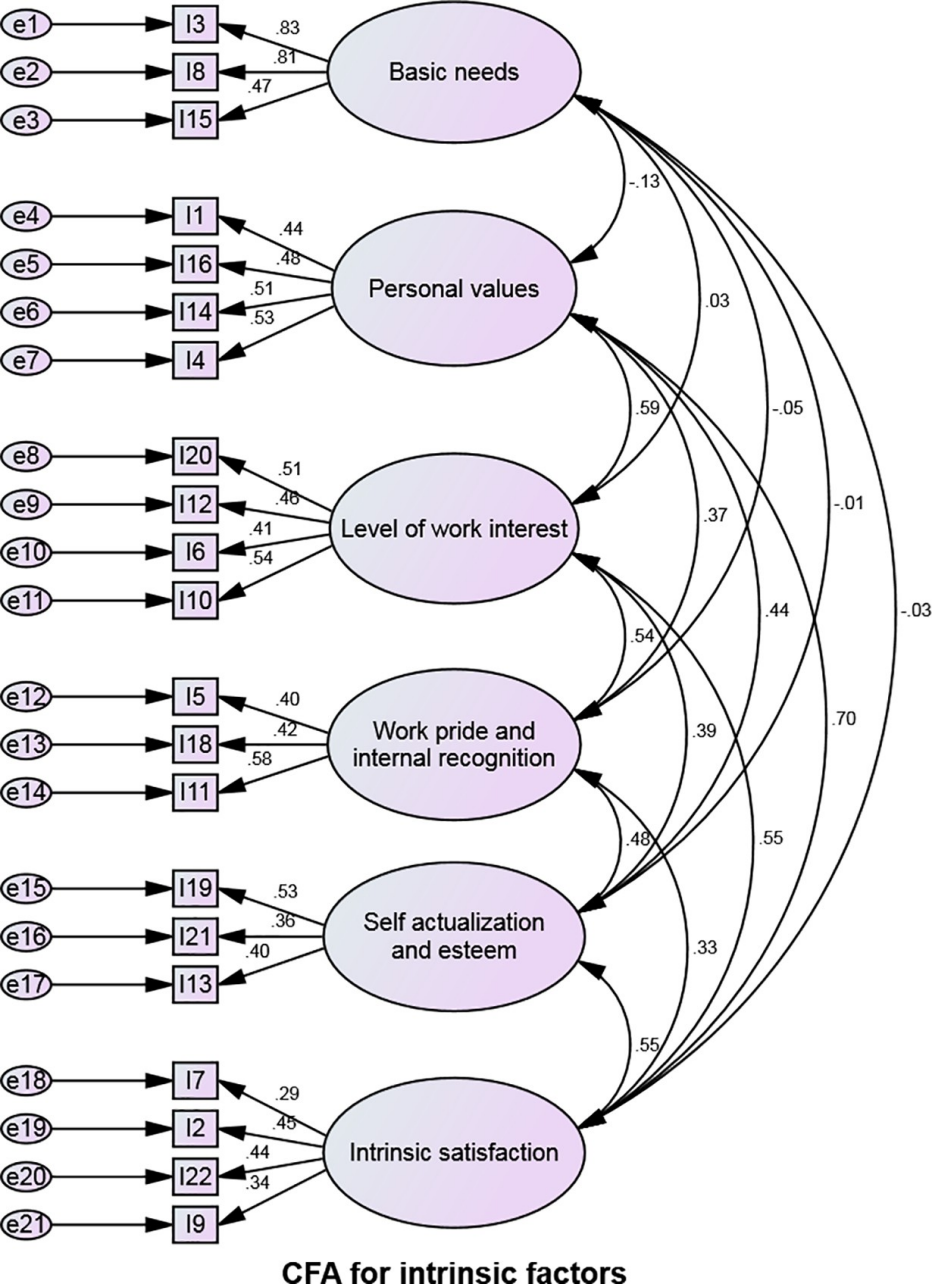
Confirmatory Factor Analysis (CFA) for intrinsic factors.

**Fig 3:**
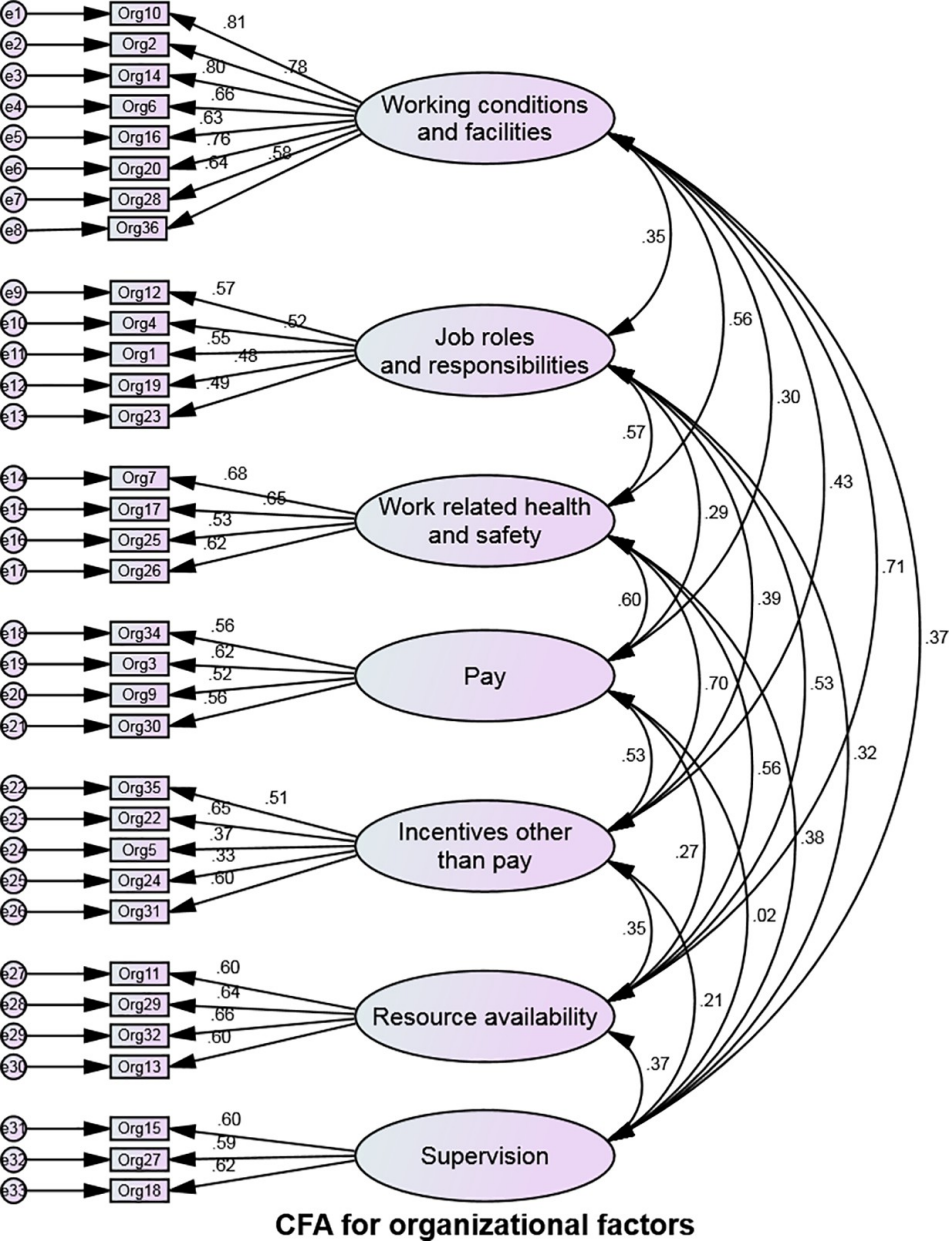
Confirmatory Factor Analysis (CFA) for organizational factors.

**Fig 4:**
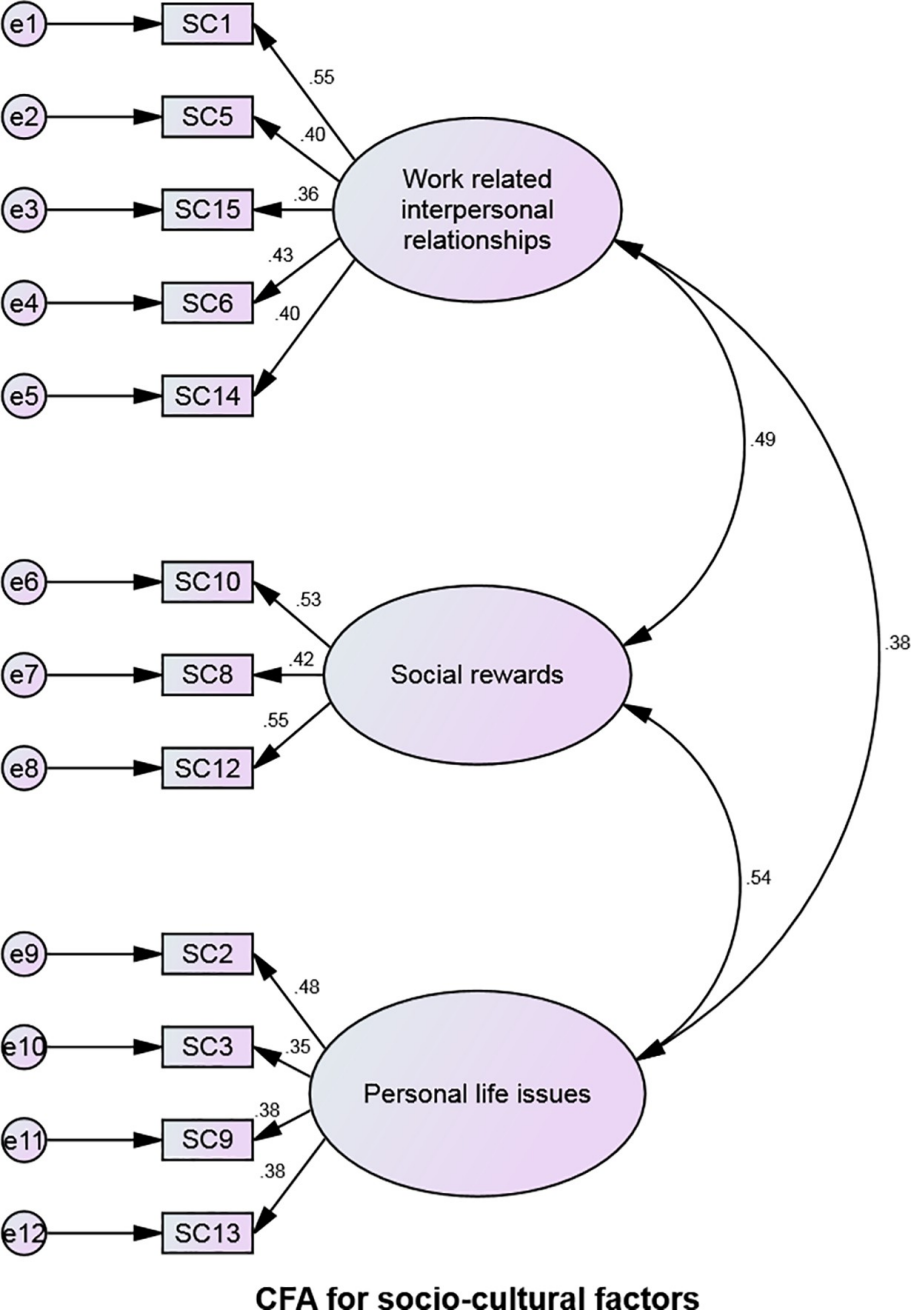
Confirmatory Factor Analysis (CFA) for socio-cultural factors.

Confirmatory factor analyses for intrinsic, organizational and socio-cultural sections of the questionnaire are shown in Figs 2–4. Modification indices were consulted to determine if there was an opportunity to improve the model. Table 4 below indicates that the goodness of fit for our measurement model, which was found to be sufficient for all three sections of the questionnaire [57].

**Table 4:** Fit indexes for intrinsic, organizational & socio-cultural questionnaires.

Metric	Observed value			Recommended*
	Intrinsic	Organizational	Socio-cultural	
**Normed Chi-square (cmin/df)**	1.061	1.423	1.415	<3 Good, <5 permissible
**GFI**	0.957	0.902	0.968	>0.95 Great, >0.9 Permissible
**CFI**	0.986	0.936	0.920	>0.95 Great, >0.9 Good, >0.8 Permissible
**RMSEA**	0.013	0.034	0.033	<0.050 Good, 0.50–0.1 Moderate
**PCLOSE**	1.00	1.00	0.937	>0.05

• The thresholds listed in the table are taken from Hu and Bentler. Hu Lt, Bentler PM: Cutoff criteria for fit indexes in covariance structure analysis: Conventional criteria versus new alternatives. *Structural equation modeling*: *a multidisciplinary journal* 1999, 6(1):1–55.

## Discussion

This study showed various known intrinsic, organizational and socio-cultural determinants of HCP motivation. Although literature shows overlapping and complexity in categorization of different individual items into intrinsic, organizational and socio-cultural factors (Tables 1–3), it was found that several needs may be operating concurrently. In addition, experts’ opinion were continuously followed to minimize mismatch in study settings. Intrinsic factors identified were; basic needs, work interest, personal values, work pride and recognition, self-actualization and esteem and intrinsic satisfaction. Organizational factors recognized were; working conditions, job roles and responsibilities, work related health and safety, salaries and other financial incentives, resources availability, supervision and other non-financial incentives including training and higher qualification opportunities. In terms of socio-cultural factors, including relationships, personal life issues and social recognition were identified. The results of the factor analysis also emphasized the importance of these factors among study physicians.

As with most tools, the possibility of response bias (e.g. social desirability) may exist. In an effort to reduce this possibility, the instrument used no negatively worded items and local experts were involved throughout the process of tool development and analysis. Additionally, the self-administered questionnaires were returned anonymously. Another limitation of the study is the age bias of the respondents. Many of the physicians who participated in the study were younger and early in their career, which could have affected the outcome of the factor analysis. Nevertheless, the instrument is still applicable in this region, as the majority of practicing physicians in Pakistan are young, and the risk of emigration from rural to urban or to developed countries is the highest among this age group, since they are in the early stages of their career and are generally more mobile [25]. A recommendation would be to oversample older, more established physicians to test the tool's applicability further. Moreover, the dynamic and complex nature of motivation may limit the findings obtained from self-administered scales completed at a given time point and may suggest for longitudinal or frequently repeated studies also. Lastly, the lack of other tools from developing countries and particularly from the region, related to HCP motivation did not allow the comparison of the performance of this tool against others. In developing countries, the issues associated with poor quality of care and health system management are expected to worsen, given current health worker shortages and the problem of HCP migration [25]. In Pakistan, at least five to six thousand doctors have left the country in the last five years, although this figure is believed to be an underestimation due to limited availability of data [58]. Physicians are eager to seek out opportunities to further their training, earn higher salaries, work in more stable environments and take advantage of career opportunities that exist in urban settings or developed countries. The large investments made by developing countries to educate and train physicians are lost if workers immigrate to other regions [25]. Low motivation is a powerful push factor for migration that researchers and policy makers need to tackle to retain qualified health personnel and strengthen health systems [59, 60]. The development of reliable and valid tools to assess motivation in developing countries is one of the first steps required to address these issues. Though solid instruments exist for use in developed countries [6, 11, 18], little exists for developing countries [20, 21, 32, 35].

Study findings provide evidence to support different aspects of Maslow's, Herzberg’s and ERG theories [44–46]. In addition, salaries, workplace conditions and facilities, and supervision were also listed as important motivators, which may indicate that their absence may lead to dissatisfaction among physicians. At the same time, the ERG theory asserts that more than one need can be present in an individual at any one time [44]. The list of factors associated with physician motivation in this study also lends support to this theory. Motivation is intrinsically associated with satisfaction [61, 62]. Motivated physicians are assumed to be more satisfied and to perform better. While motivation and satisfaction might not be directly visible, yet both are significant for health workers retention and performance [5, 63].

Overall, factors found in the study also support the findings of prior research that money or financial incentives are not the only factors important for HCP motivation in other low and middle income countries [11, 14, 64, 65]. Poor working conditions and the quality of care provided to patients can also greatly affect job satisfaction [25, 66]. A lack of appropriate resources can also lead to frustration and demotivation due to compromised health care quality, which can occur despite the intentions or capabilities of health providers [19, 52, 67]. Other determinants such as; lack of infrastructure and staff residence [68], professional growth [68, 69], lack of appreciation [69], job description [70] were also reported in other studies. Training and higher qualification prospects have been associated with higher motivation [62] and satisfaction [71]. Social rewards such as respect and serving community [72–74] as well as recognition from communities and employers have also been found to be a fundamental motivating factor for health workers [23, 65, 75].

In Pakistan, fewer studies conducted on health providers’ motivation and/or job satisfaction have also found many factors that are comparable to the tool findings. Mostly studies have explored the issue in general using tools previously used in other developed countries or dissimilar settings with no or different categorization of determinants [22, 76, 77]. Presumably, in context of developing and exploring tool to assess motivation across intrinsic, organizational and socio-cultural level, this study is first of its kind in Pakistan. Determinants identified in other relevant studies were; working conditions [22, 25, 77], pay and other financial incentives [22, 76, 77], work load, professional and career growth opportunities [22, 27, 76], lack of basic amenities [77], job description, supervision and security [22]. Although, many factors can play parts, workforce shortage might affect work load and resource limited settings may offer inferior working conditions and pay. Yet the magnitude to which each determinant can affect different individuals’ motivation and/or satisfaction in different settings remains complex. This dilemma might be attributed to the possible inter-relatedness of various determinants and dynamic nature of motivation and satisfaction which may require continuous or frequent exploration. Besides alignment with acknowledged and recent literature from the region, this tool showed acceptable reliability and validity, consistent with other tools developed and used in other low and/or middle income settings [20, 21, 32, 35]. This questionnaire is relatively comprehensive in approach and suitable to assess motivation and job satisfaction in resource limited settings. Furthermore, CFA findings showed good model fit which suggests it is appropriate for use in similar settings. However, testing the applicability of the instrument among other HCPs on larger scale, in similar and diverse settings and along with qualitative approaches is recommended to round out our understandings.

## Conclusion

In addition to the development of a valid and reliable tool to explore motivation, this study identified important intrinsic, socio-cultural and organizational factors behind physician motivation and job satisfaction in this region. Considering limited studies and tools developed and tested on this issue in the region, study findings can potentially assist managers and policy makers to identify feasible determinants to deal with in resource limited settings like Pakistan. Findings also highlight the need for further investigations across different health facilities to assist in policy planning and interventions, particularly in low and middle-income countries.

## Supporting information

S1 Data (SAV)

S1 Questionnaire (PDF)
