# Supplementary material for: Developing and assessing a tool to measure motivation among physicians in Lahore, Pakistan
Source: PLoS One. 2018 Dec 31;13(12):e0209546. doi: 10.1371/journal.pone.0209546 (PMC6312213; doi:10.1371/journal.pone.0209546)
Supplement: S1 Questionnaire — (PDF) [file pone.0209546.s002.pdf]

Health Care Provider Motivation (HCP):
Questionnaire: for this part of the study

- **A- Demographic and general information**
- **B- Factors related to Motivation**
  1. **Intrinsic**
  2. **Organizational**
  3. **Socio-cultural factors**

A- Demographic and general information:

This section involves questions related to demographics, background and general personal information. The questions with no options given have to be filled in and where options are given, reply has to be marked tick/cross.

1. Age: ------ years
2. Gender: **1**.❑ Male **2**.❑ Female
3. Marital status: **1**.❑ Single **2**.❑ Married **3**.❑ Widowed **4**.❑ Divorced
4. Name of the Health facility: **1**. ❑BHU/RHC **2**.❑THQ/DHQ **3**.❑FMH **4**.❑ MAYO
5. Health facility level: **1**.❑ Primary **2**.❑ Secondary **3**.❑ Tertiary
6. Category of health facility in Health Sector: **1**.❑ Public **2**. ❑ Private **3**.❑ Both
7. Current Designation: ---------------
8. Type of job: **1**.❑ full time **2**.❑ part time
9. Working Hrs: -------hrs/wk
10. Work category: **1**.❑ Indoor **2**.❑ Outdoor **3**.❑ Emergency **4**.❑ On call **5**.❑ mix(specify) -----
11. How long have you been working in this health facility: ----------years/months
12. Qualification: **1**.❑ Graduation (MBBS) **2**.❑ Post graduation (FCPS, FRCS, Dipl. etc.)

Directions for Intrinsic, organization and socio-culture sections:
Use the scale below to give opinion for each statement. Focus is on how you perceive for each statement in your current job and how important is that generally to your motivation. These two options are asked in the columns next to the statements. In first columns next to statements answers will be given using the scales to indicate how much you agree or disagree with the statement by placing the number that best corresponds to your answer. Scale ranges from 1- Strongly disagree to 5- Strongly agree.

In second columns next to statements answers will be given using scales to indicate how much you give importance to the statement by placing the number that best corresponds to your answer. Scale ranges from 1- Unimportant to 5- Very important.
Note: there is no right or wrong answers, just respond how you perceive.

	**Intrinsic:**	
	How much you agree or disagree with the statements below in your current job	Strongly disagree 1<br>Disagree 2<br>Neither 3<br>Agree 4<br>Strongly agree 5
1.	I feel interested in my current job	1 2 3 4 5
2.	I like to work hard during my work	1 2 3 4 5
3.	I feel motivated with the availability of drinking water during work	1 2 3 4 5
4.	I feel motivated with the challenging work given to me	1 2 3 4 5
5.	I feel motivated with my status	1 2 3 4 5
6.	I feel motivated being able to work ethically in general	1 2 3 4 5
7.	I feel motivated to be dependable and reliable	1 2 3 4 5
8.	I feel motivated with the availability of adequate food during work	1 2 3 4 5
9.	I feel motivated with my work efficiency	1 2 3 4 5
10.	I feel motivated with my sense of accomplishment during work	1 2 3 4 5
11.	I feel motivated with the pride that I get being a part of this hospital	1 2 3 4 5
12.	I get self-respect from my work	1 2 3 4 5
13.	I feel motivated with the recognition given to me for good work	1 2 3 4 5
14.	Work is meaningful to me	1 2 3 4 5
15.	I feel motivated with the availability of proper rest room for long and night duties	1 2 3 4 5
16.	I like my work	1 2 3 4 5
17.	I feel motivated with the amount of functional independence given to me during work	1 2 3 4 5
18.	I feel motivated with importance given to me by hospital management	1 2 3 4 5
19.	I feel motivated with the amount of variety in my work	1 2 3 4 5
20.	I take my work as a source of social respect	1 2 3 4 5
21.	I feel motivated with creativity opportunities during work	1 2 3 4 5
22.	I feel motivated with my work in general	1 2 3 4 5

	**Organizational:**	
	How much you agree or disagree with the statements below in your current job	Strongly disagree 1<br>Disagree 2<br>Neither 3<br>Agree 4<br>Strongly agree 5
1.	I am satisfied with the availability of proper toilets and hand washing facilities in work place	1 2 3 4 5
2.	I am satisfied with the availability of proper dressing rooms in work place	1 2 3 4 5
3.	I am satisfied with the availability of proper hygienic and serviceable conditions	1 2 3 4 5
4.	I am satisfied with the availability of proper lighting during work	1 2 3 4 5
5.	I am satisfied with the clean and maintained work place	1 2 3 4 5
6.	I am satisfied with the availability of adequate designated work area	1 2 3 4 5
7.	I am satisfied with the availability of proper ventilation in work place	1 2 3 4 5
8.	I am satisfied with my work load	1 2 3 4 5
9.	I am satisfied with the general working environment to accomplish quality job	1 2 3 4 5
10.	I am satisfied with the amount of safety during work from diseases like HIV/AIDS, T.B, and Hepatitis etc.	1 2 3 4 5
11.	My hospital provides me measures for safety during work from diseases like HIV/AIDS, T.B, and Hepatitis etc.	1 2 3 4 5
12.	I am satisfied in general with the occupational health	1 2 3 4 5
13.	I am satisfied with the insurance paid for me	1 2 3 4 5
14.	I am satisfied with my job description	1 2 3 4 5
15.	I am satisfied with the referral procedures	1 2 3 4 5
16.	I work according to my job description	1 2 3 4 5
17.	I am satisfied with the clarity of my roles and responsibilities	1 2 3 4 5
18.	I am satisfied with the clarity of roles and responsibilities of different HCPs	1 2 3 4 5
19.	I am satisfied with the pension planned for me	1 2 3 4 5
20.	I am satisfied with the allowances I get	1 2 3 4 5
21.	I am satisfied with the opportunities for career advancement and growth given to me	1 2 3 4 5

22.	I am satisfied with the opportunities for promotion available to me	1 2 3 4 5
23.	I am satisfied with the opportunities for higher qualification available to me	1 2 3 4 5
24.	I am satisfied with my job security	1 2 3 4 5
25.	I am satisfied with overall supplies available during work	1 2 3 4 5
26.	I am satisfied with the drugs available during work	1 2 3 4 5
27.	I am satisfied with the equipment available during work	1 2 3 4 5
28.	I am satisfied with the number of staff available during work	1 2 3 4 5
29.	I get feedback from my supervisor	1 2 3 4 5
30.	I am satisfied with the quality of supervision I get	1 2 3 4 5
31.	Supervisors provide fair feedback for all	1 2 3 4 5
32.	I feel I have a good competitive salary for the profession I have	1 2 3 4 5
33.	I am satisfied with the subsidies I get	1 2 3 4 5
34.	I feel my income is in accordance to my education, skills and knowledge	1 2 3 4 5
35.	I am satisfied with increments in my salary	1 2 3 4 5
36.	I get enough pay to fulfill basic needs like food, accommodation, and transport for me and my family	1 2 3 4 5

	**Socio-cultural:**	
	How much you agree or disagree with the statements below in your current job	Strongly disagree 1<br>Disagree 2<br>Neither 3<br>Agree 4<br>Strongly agree 5
1.	I feel motivated if co-workers help each other	1 2 3 4 5
2.	I feel motivated with the personal support I get from co-workers when required	1 2 3 4 5
3.	I am satisfied with my personal life issues	1 2 3 4 5
4.	I am satisfied with the team work around me during work	1 2 3 4 5
5.	I feel motivated if co-workers respect each other	1 2 3 4 5
6.	I feel motivated with good relationship between doctors and nurses	1 2 3 4 5
7.	My work affects my other priorities like responsibilities at home, visiting friends, pursuing a hobby etc.	1 2 3 4 5
8.	I feel motivated with the feedback I get from the patients	1 2 3 4 5
9.	I feel motivated the way hospital supports and respects my personal life issues	1 2 3 4 5
10.	I feel motivated with the respect I get from patients	1 2 3 4 5
11.	I prefer to work in a team	1 2 3 4 5
12.	I feel motivated with the respect I get from the community	1 2 3 4 5
13.	I feel motivated with the feedback I get from the community	1 2 3 4 5
14.	I feel motivated if co-workers willingly share expertise and skills with other colleagues	1 2 3 4 5
15.	I am satisfied with my general interpersonal relations during work	1 2 3 4 5

Thanks for your precious time and input.
